# The association between endometrial thickness and pregnancy outcome in gonadotropin-stimulated intrauterine insemination cycles

**DOI:** 10.1186/s12958-019-0455-1

**Published:** 2019-01-23

**Authors:** Yiwen Liu, Xiang Y. Ye, Crystal Chan

**Affiliations:** 1 Faculty of Medicine, University of Toronto, 1 King’s College Circle Medical Sciences Building, Room 2109, Toronto, ON M5S 1A8 Canada; 2 Maternal Infant Care Research Centre, Mount Sinai Hospital , 700 University Ave, Toronto, ON M5G 1X6 Canada; 3 Department of Obstetrics and Gynaecology, University of Toronto, 123 Edward St. Suite 1200, Toronto, ON M5G 1E2 Canada; 4 Mount Sinai Fertility, Mount Sinai Hospital, 250 Dundas St W #700, Toronto, ON M5T 2Z5 Canada

**Keywords:** Endometrial stripe thickness, Intrauterine insemination, Injectable gonadotropins, Clinical pregnancy, Pregnancy outcome

## Abstract

**Background:**

Intrauterine insemination (IUI) is the first-line treatment for non-tubal infertility. Injectable gonadotropins are often chosen as adjunctive stimulation to promote the growth of ovarian follicles in IUI cycles. The growing follicles produce estrogen, which induces endometrial proliferation and increased endometrial stripe thickness (EST). The association between EST and pregnancy outcome in gonadotropin stimulated IUI is not well studied. The objective of this study is to determine if EST can predict pregnancy outcome in gonadotropin-stimulated IUI cycles.

**Methods:**

A retrospective review was conducted of all exclusively gonadotropin-stimulated IUI cycles performed between 2012 and 2015 at an academic fertility clinic. Mean endometrial thickness was compared in positive versus negative cycles using Student T-test. Peak EST values were then divided into four groups of < 7 mm, 7.0–10.4 mm, 10.5–13.9 mm, and ≥ 14 mm. Multiple logistic regression analysis adjusted for potential confounders was conducted to assess the impact of peak EST on cycle outcome.

**Results:**

Our sample consisted of 1065 IUI cycles representing 548 patients with a 16.9% clinical pregnancy rate and 20.5% conception rate. No significant differences in mean peak EST were observed between cycles that achieved clinical pregnancy or conception and those that did not. Division of peak EST into four groups showed a non-linear relationship between peak EST and cycle outcome, with highest rates of positive outcomes between 10.5–13.9 mm. The odds of clinical pregnancy and conception increased by 38 and 44% respectively with each subsequent peak EST category up to 10.5–13.9 mm, following which they declined.

**Conclusion:**

This is the largest study to date evaluating the effect of peak EST on gonadotropin-stimulated IUI cycles exclusively. The lack of significant difference in peak EST between positive and negative outcomes cycles may be due to the non-linear relationship between cycle outcomes and peak EST. Peak EST in the range of 10.5–13.9 mm was associated with significantly higher conception rates and a trend towards higher clinical pregnancy rates.

This non-linearity is likely one of the reasons that EST in isolation was found to be a poor predictor of IUI outcomes, and therefore is not appropriate to be used as the sole indicator for cycle cancellation.

## Background

Intrauterine insemination (IUI) is the first-line treatment for couples with unexplained or mild male factor infertility. During an IUI cycle, adjunctive stimulation can be achieved via clomiphene citrate (CC), aromatase inhibitors, or injectable gonadotropins to promote the growth of ovarian follicles. Estrogen produced by growing follicles induces endometrial proliferation, leading to increased thickness of the endometrial stripe as measured by transvaginal ultrasound. Peak endometrial stripe thickness (EST) has been studied as a predictive factor of pregnancy outcome in IUI, but most studies have focused on CC-stimulated cycles. This is due to the fact that CC is a selective estrogen receptor modulator known to have anti-estrogenic activity at the level of the endometrium, where it has been shown to reduce uptake of radioactive estradiol by the uterus, and thus may attenuate the endometrium quantitatively and qualitatively [1, 2]. Several studies have demonstrated that EST is lower in CC-stimulated cycles compared to natural cycles [1, 3, 4].

In CC-stimulated cycles, this decline in EST may have negative effects on endometrial receptivity and pregnancy outcomes. Esmailzadeh et al. demonstrated that peak EST was greater in CC-induced IUI cycles that resulted in pregnancy versus cycles that did not (10.1 +/− 3.0 vs. 7.7 +/− 3.5) [5]. Similarly, Warrington, Faraj & Willett quantified that for every millimeter increase in endometrial thickness, the odds of conception increased 14% when controlling for potential confounders among CC induced IUI cycles [6].

Unlike CC, injectable gonadotrophins do not alter estrogen receptor kinetics nor attenuate the endometrial lining in the same way. Peak EST has been shown to be thicker in gonadotropin-stimulated compared to CC-stimulated cycles [7]. Therefore, the same level of thin EST may not be comparable between clomid and FSH cycles. Indeed, thin FSH lining may indicate pathological differences compared to a thin clomid EST.

However, the association between peak EST and pregnancy outcome in gonadotropin-stimulated IUI has been much less examined. Several studies have studied patients stimulated with a combination of CC and gonadotropins and demonstrated variable conclusions about peak EST and pregnancy outcomes [8–10].

A recent meta-analysis by Weiss et al. examining EST in IUI cycles found no evidence that EST was associated with chances of clinical pregnancy [11]. However, this study encompassed a heterogeneous mixture of ovarian stimulation methods including CC, letrozole, and injectable gonadotropins. In addition, data comparing EST between cycles that were pregnant vs negative were only available for a subset of the studies included for analysis.

There are few studies looking at gonadotropin stimulation alone, without the confounding influence of CC. Yavuz et al. demonstrated a higher clinical pregnancy rate in gonadotropin-stimulated IUI cycles when peak EST was ≥8 mm, but pregnancy rates were lower in this study than usually quoted (5.8% if EST ≥ 8 mm vs. 2.5% if EST < 8 mm) [12]. However, other studies on gonadotropin-IUI showed no significant association between peak EST and pregnancy outcomes [11, 13, 14].

The objective of this current study is to assess for an association between peak EST and pregnancy outcome in gonadotropin-stimulated IUI cycles. To our knowledge, this is the largest study to date on exclusively gonadotropin-stimulated IUI cycles and will help guide management of this common treatment.

## Material and methods

### Study population and participants

This was a retrospective cohort study approved by the Mount Sinai Hospital Research Ethics Board. Women who underwent exclusively gonadotropin-stimulated IUI at Mount Sinai Fertility Clinic between January 1, 2012 and Dec 31, 2015 were included. Inclusion criteria included patients with at least one patent fallopian tube as determined via saline sonohysterogram or hysterosalpingogram. In addition, semen analysis demonstrating a total motile sperm count of at least one million, and peak EST measurement within 36 h of LH surge or ovulation trigger were required for cycles to be eligible in this study.

### Ovarian stimulation and IUI protocol

Ovarian stimulation was started on either a spontaneous day-3 in regularly cycling patients or an “assigned” day-3 in oligomenorrheic patients. Patients with hypothalamic hypogonadism were treated with a combination of highly purified hMG (Menopur, Ferring, Canada) and recombinant FSH (Gonal F, EMD Serono or Puregon, Merck, Canada), while those with unexplained infertility/PCOS were treated with only FSH. In addition to hormonal profile, dosages were determined by patient age, body mass index, ovarian reserve assessment and history of previous response.

Cycle monitoring was performed with transvaginal ultrasound (TVUS) and serum estradiol/LH/progesterone measurements. After the patient had voided, TVUS was performed for ovarian follicle and EST measurement. EST was measured in the sagittal plane from the outer edge of the endometrial-myometrial interface on one side to the other, at the widest part of the endometrium. All TVUS measurements were conducted with a 5MHZ vaginal transducer (Toshiba).

If a spontaneous LH surge occurred during the cycle, the surge was supplemented with subcutaneous injection of 250 μg of recombinant hCG (Ovidrel, EMD Serono, Canada), and a single IUI was performed 24 h after detection of the surge. If no spontaneous LH surge was detected and the lead follicle(s) were ≥ 18 mm, ovulation was triggered with 250 μg of subcutaneous recombinant hCG and the IUI was performed 36 h after trigger. Luteal phase support was provided with micronized progesterone 200 mg vaginal suppositories nightly.

### Outcome assessment

The primary outcome was clinical pregnancy, defined as the presence of a gestational sac at 6.5–7.5 weeks of gestation by TVUS. A secondary outcome measure was conception, as indicated by positive beta-hCG two weeks after IUI.

### Statistical analysis

The study population was summarized descriptively. The patient characteristics were compared between positive clinical pregnancy versus negative cycles using Chi-square test for categorical variables and Student T test for continuous ones. To examine the association between the peak EST and the primary outcome, we first compared peak EST between cycles achieving clinical pregnancy and negative cycles using Student T test. A univariate linear regression model with generalized estimating equation (GEE) approach was used to account for the clustering of cycles within the same patient. Multiple logistic regression model with GEE approach adjusted for potential confounders identified based on the literature and the univariate analyses was also conducted to assess the impact of peak EST on the primary outcome.

To further determine the association between peak EST and clinical pregnancy, we divided peak EST into four groups using three cut-off values of 7, 10.5 and 14: < 7 mm, 7.0–10.4 mm, 10.5–13.9 mm, and ≥ 14 mm. The cut-offs of 7 and 14 were chosen based on the distribution of peak EST and the previous literature [15–17] while the cut-off of 10.5 was the median of 7 and 14. The patient characteristics were then compared among the four peak EST groups to assess the relationship between the characteristics and EST using Chi-square test for categorical variables and F-test for continuous ones.

To examine the relationship between peak EST and the primary outcome, we estimated the rate of the primary outcome in cycle level for each EST group and compared the rates among the four groups using Chi-square test. The Cochran-Armitage trend test was conducted to examine the trend in the clinical pregnancy rates with respect to the peak EST. Multivariable piecewise generalized linear regression was conducted for the outcome adjusted for the potential confounders to examine its non-linear relationship with peak EST. We also conducted multiple logistic regression analysis to compare the clinical pregnancy among the four groups, adjusted for the potential confounders. For both piecewise linear regression and logistic regression, the GEE approach was used to account for the clustering of cycles within the same patient. Similar methods as described for the primary outcome were used to examine the relationship between peak EST and the secondary outcome of conception.

Lastly, the area under receiver operating curves (ROC) was used to assess the predictive power of peak EST for clinical pregnancy.

Data management and all statistical analyses were performed using SAS 9.3 (SAS Institute, Inc., Cary, NC). A two-sided *p*-value of < 0.05 was used to determine statistical significance.

## Results

1127 gonadotropin-stimulated IUI cycles were completed between 2012 to 2015. 8 cycles were excluded because peak EST was not measured within 36 h of LH surge or ovulation trigger. In addition, 34 cycles were removed from the study due to lack of EST measurements and 20 were removed due to lack of follow-up on pregnancy outcome.

Our final sample consisted of 1065 cycles representing 548 patients. Approximately half of these patients (49.8%) completed one cycle only. 218 (20.5%) cycles resulted in conception, including 180 (16.9%) cycles that developed into clinical pregnancy (Table 1). Among the clinical pregnancies, 24 (13.3%) cycles were twin gestations as defined by the presence of two gestational sacs on TVUS, with one additional cycle resulting in a triplet gestation.

**Table 1:** Comparison of Baseline Characteristics

Characteristics	Clinical Pregnancy			Conception		
	Positive (*n* = 180)	Negative (*n* = 885)	*P*-value	Positive (*n* = 218)	Negative (*n* = 847)	*P* value
Age	36.1 (±3.8)	37.1 (±3.8)	0.002	36.3 (±3.8)	37.1 (±3.8)	0.007
BMI						0.70
Underweight (< 18.5) Normal (18.5–24.9) Overweight (25.0–29.9) Obese (> 30)	4 (2.2%) 78 (43.3%) 38 (21.1%) 38 (21.1%)	13 (1.5%) 469 (53.0%) 185 (20.9%) 159 (18.0%)	0.69	6 (2.7%) 90 (41.3%) 52 (23.8%) 47(21.9%)	11 (1.3%) 457 (54.0%) 171 (20.2%) 150 (17.7%)	
Infertility Diagnosis						< 0.001
Male Factor Ovulatory Dysfunction Unexplained Other	40 (22.2%) 50 (27.8%) 36 (20.0%) 62 (34.4%)	193 (21.8%) 161 (18.2%) 199 (22.5%) 371 (41.9%)	< 0.001	45 (20.6%) 62 (28.4%) 47 (21.6%) 73 (33.5%)	188 (22.2%) 149 (17.6%) 189 (22.3%) 354 (41.8%)	
Anti-Mullerian Hormone (pmol/L)	14.8 (±17.6)	14.5 (±18.9)	0.87	16.0 (21.7)	14.2 (± 17.9)	0.31
Antral Follicle Count	14.5 (±9.7)	13.0 (±9.1)	0.08	14.3 (±9.74)	13.0 (±9.10)	0.10
Total Motile Sperm Count (millions)	18.7 (±11.9)	17.1 (±12.4)	0.099	19.2 (±13.2)	16.9 (±12.0)	0.02
Total FSH Dose	993.5 (±675)	1123.9 (±858)	0.059	1002.1 (± 653)	1127.7 (± 870)	0.050
Number of Dominant Follicles (≥ 15 mm)	2.5 (±1.3)	2.1 (±1.2)	0.002	2.44 (±1.29)	2.11 (±1.20)	0.001
Peak Estrogen Level (pmol/L)	2967 (±1507)	2706 (±1493)	0.035	2928 (±1458)	2701 (±1506)	0.05
Peak EST (mm)	9.54 (±1.78)	9.36 (±1.89)	0.235	9.55 (±1.83)	9.35 (±1.88)	0.159

Differences in factors previously identified as significantly impacting pregnancy outcome between clinically pregnant and negative cycles. All continuous variables are expressed as mean ± SD

**Fig. 1:**
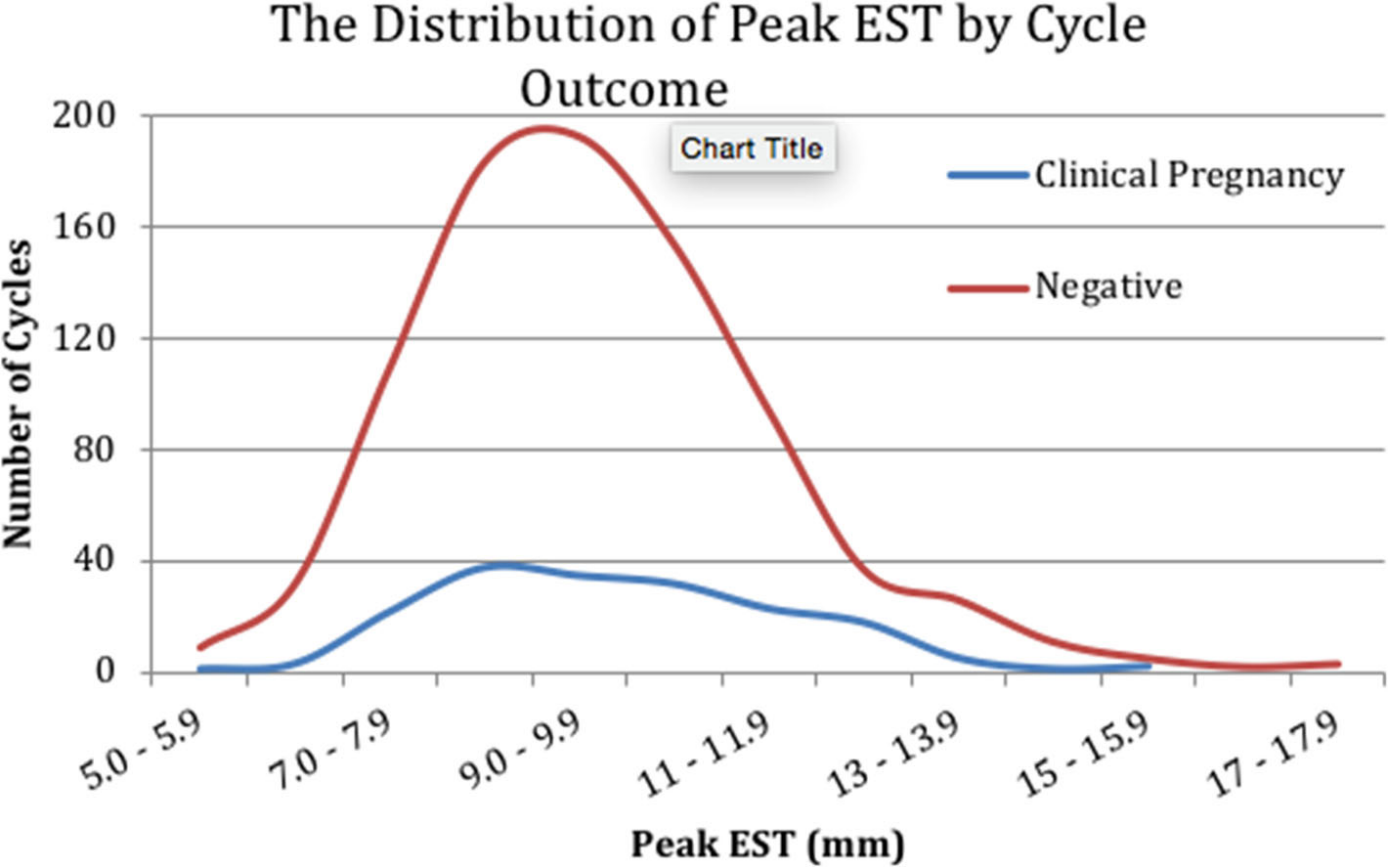
The Distribution of Peak EST by Cycle Outcome. Distribution of endometrial thickness in clinical pregnancy and negative cycles

The range of the peak EST was from 5 mm to 20 mm, and the distribution of peak EST was similar between the clinically pregnant and negative outcome cycles (Fig. 1). No pregnancies occurred when peak EST was < 5.1 mm or > 15 mm.

Patients who achieved clinical pregnancy were younger (36.1 ± 3.8 vs. 37.1 ± 3.8, *p* = 0.002), produced more dominant follicles (2.5 ± 1.3 vs. 2.1 ± 1.2, p = 0.002), and had higher peak estrogen levels (2967 ± 1507 pmol/L vs. 2706 pmol/L, *p* = 0.035), compared to their non-pregnant counterparts. No significant difference in mean EST was observed between cycles that resulted in clinical pregnancy and those that were negative (*p* = 0.235). Similarly, no significant difference in mean peak EST was observed in those who conceived compared to those who did not (9.55 mm vs 9.35 mm, *p* = 0.159) (Table 1).

From the univariate regression for both outcomes, age was found to be significantly and inversely associated with likelihood of conception and clinical pregnancy. The likelihood of conception was marginally, but significantly, increased when the total motile sperm count was increased (OR 1.01, 95% CI 1.003–1.025). Both peak estrogen level and number of dominant follicles were independently associated with conception and clinical pregnancy, but given their collinearity, only the latter was included in the multivariate model to avoid multiple correlated dependent variables. All other factors were found to be not significant in relation to clinical pregnancy or conception outcome (Table 2).

**Table 2:** Association between factors affecting pregnancy and IUI cycle outcome

Characteristics	Clinical Pregnancy	Conception
	OR (95%CI)	OR (95%CI)
Peak EST, (0.1 mm)	1.63 (0.71, 3.77)	1.74 (0.80, 3.79)
Age	0.94 (0.90, 0.98)	0.95 (0.91, 0.99)
BMI	1.01 (0.98, 1.03)	1.01 (0.99, 1.04)
Peak Estrogen/1000	1.12 (1.01, 1.24)	1.10 (1.00, 1.21)
Dominant Follicles	1.23 (1.08, 1.39)	1.23 (1.10, 1.39)
Antral Follicle count	1.02 (0.999, 1.034)	1.01 (0.998, 1.03)
AMH	1.00 (0.99, 1.01)	1.01 (0.996, 1.013)
Motile Sperm count	1.01 (0.998, 1.023)	1.01 (1.003, 1.025)

The likelihood of clinical pregnancy or conception outcome according to univariate analysis of factors previously identified as impacting IUI cycle outcome

**Fig 2:**
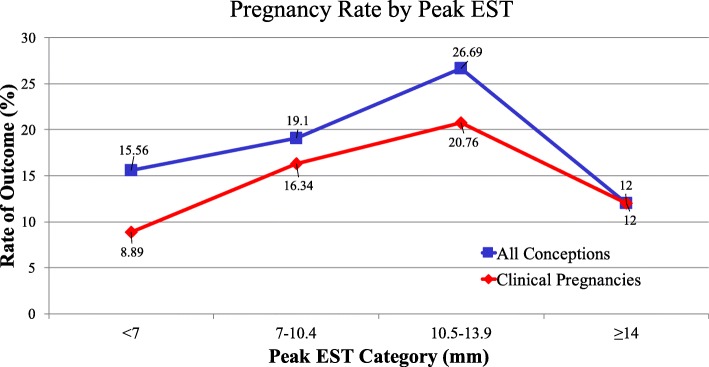
Pregnancy Rate by Peak EST. Both clinical pregnancy and conception outcome rates increased with respect to the endometrial thickness categories (Cochran-Armitage trend test: *p* < 0.04) when peak EST < 14 mm

To further examine the association between peak EST and cycle outcomes, peak EST was classified into four groups based on peak EST distribution in our data and previously published literature [15–17]: < 7.0 mm (Group 1), 7.0–10.4 mm (Group 2), 10.5 mm–13.9 mm (Group 3) and ≥ 14 mm (Group 4). The number of cycles in each group was 45 (4.2%), 759 (71.2%), 236 (22.2%) and 25 (2.4%) respectively. The rates of both clinical pregnancy and conception increased with ascending peak EST category up to 14 mm (trend test: *p* < 0.04), after which they decreased (Fig. 2). This suggests an ‘n’ shaped or nonlinear relationship between the rate of both outcomes and peak EST. This distribution was somewhat unexpected, as previous studies had focused on the detrimental effect of decreased EST on cycle outcome, and not when the lining was thickened.

To further delineate this, piecewise generalized linear regression models for the outcomes were conducted, adjusting for potential confounders. The results showed that the odds of clinical pregnancy increased by 38% (=exp. (0.32)-1, *p* = 0.06) when peak EST increased from a category group to the next higher one before EST < 14 mm, while the odds decreased by 60% (=exp.(− 0.94)-1, *p* = 0.17) when peak EST decreased from group 3 to group 4. The odds of conception increased by 44% (exp(0.37)-1, *p* = 0.02) when the EST increased from a category to the next higher one before EST < 14 mm, and the odds of conception decreased by 72% (=exp.(− 1.27)-1, p = 0.06) when the EST decreased from group 3 to group 4 (Table 3).

**Table 3:** Comparison of outcomes among Peak EST groups for all patients treated

Models:		Outcomes			*P*-value
		Clinical Pregnancy	*P*-value	Conception	
		estimate (se)		estimate (se)	
Piece-wise linear Model**	Peak EST (peak EST < 14)	0.32 (0.17)	0.06	0.37 (0.16)	0.022
	Peak EST (Peak EST > =14)	−0.94 (0.69)	0.17	−1.27 (0.68)	0.06
Multiple logistic regression model ***		Adjusted OR(95%CI)	*P*-value	Adjusted OR(95%CI)	*P*-value
	Group 1 vs 3	0.44(0.16,1.21)	0.11	0.61(0.23,1.58)	0.31
	Group 2 vs 3	0.77(0.52,1.14)	0.2	0.69(0.48,0.99)	0.046
	ref, Group 3	1		1	
	Group 4 vs 3	0.6(0.19,1.94)	0.39	0.46(0.15,1.38)	0.16

**Multivariable piecewise linear model with change point at peak EST = 14

***: The adjusted OR (95%CI) were determined based on Multiple logistic regression model for the outcomes with GEE approach to account for the clustering of cycles for the same patient

For both models, the potential confounders adjusted for were age, dominant follicles, motile sperm count, and peak estrogen

Group 1: peak EST < 7 (mm); Group 2: peak EST: 7–10.4 (mm); peak EST: 10.5–13.9 (mm); Group 4: peak EST > =14 (mm)

To be conservative, we also conducted a multiple logistic regression analysis adjusted for potential confounders to compare both outcomes among the four peak EST groups. The results (Table 3) were consistent with the ones using piecewise generalized linear regression. Group 3 was chosen as the reference variable because it contained the highest rates of positive outcomes and therefore represented the most optimal EST state. The results showed the odds of conception at group3 (peak EST 10.5–13.9 mm) was significantly higher compared to group 2. The odds of conception at group 3 was 54, 31 and 39% higher compared to group 4, 2 and 1 respectively, although no significant effect was observed. Similarly, the odds of clinical pregnancy in group 3 increased by 40, 23 and 56% compared to group 4, 2 and 1 respectively, although this effect was not significant.

We also examined the predictive power of peak EST on clinical pregnancy outcome using ROC analysis. The area under the curve was 0.54 in predicting clinical pregnancy using peak EST (data not shown), which suggests that peak EST is a poor predictor variable for clinical pregnancy outcome.

## Discussion and conclusions

To our knowledge, this study is the largest to date examining the relationship between peak EST and gonadotropin-stimulated IUI cycle outcome. This is an important relationship to study, as clinicians managing IUI cycles have a paucity of data with which to counsel patients with a thin lining. The results of our study, which showed a clinical pregnancy rate of 8.89% when peak EST < 7 mm, do not support the practice of routinely cancelling cycles when the lining is below this threshold. The small overall number of cycles in the < 7 mm category reinforces previous findings that gonadotropins do not attenuate the endometrial lining to the same degree as CC [7].

Our first method of analysis comparing peak EST between positive (conception and clinical pregnancy) and negative IUI outcome cycles using univariate analysis showed no difference in peak EST between outcome groups. These results are in line with the recently published meta-analyses conducted by Weiss et al., which found no association between peak EST and cycle outcome in a patient population receiving a mixture of induction protocols including CC, letrozole, and gonadotropins [11].

The lack of significant results when simply comparing peak EST between outcome groups may be subject to three possible interpretations. Firstly, there may merely be no difference in peak EST between outcome groups. Secondly, statistical significance may not have been achieved due to sample size limitations, although clinically there may still be a true difference in peak EST between outcome groups. Lastly, the statistical difference may not have been observed through these initial analyses due to a non-linear relationship between peak EST and cycle outcomes. If the association between peak EST and outcomes were to be non-linear, this method of simply comparing EST between two outcome groups would not be appropriate since it would dilute the effect size. Previous literature had assumed a linear relationship between endometrial thickness and success rate, which was a limitation in their analyses [12].

Therefore, we moved on to examine the possibility of a non-linear relationship by splitting peak EST values into four groups. We examined the trend in outcome rates across the 4 EST groups, and also compared peak EST among the 4 groups. Our initial graphical exploration indicated that the highest rates for both clinical pregnancy and conception were in the peak EST category of 10.5–13.9 mm. The observation of decreased pregnancy rates at both the low and high ends of peak endometrial thicknesses was previously demonstrated by Dinelli et al., who found the highest rates of pregnancy when peak EST ranged from 10 to 11 mm [18].

Next, we conducted piecewise linear regression using the four peak EST groups using both univariate and multivariate analysis. Multivariate analysis included factors previously associated with IUI cycle outcome: the woman’s age, number of dominant follicles, peak estrogen level, and the total motile sperm count. These findings concur with previous studies that examined predictive factors of pregnancy outcome [5, 18].

Unique to our study is the discovery of a non-linear relationship between increasing peak EST and pregnancy outcomes. We demonstrated higher conception rates with each subsequent category peak EST (< 7 mm, 7–10.4 mm, and 10.5–13.9 mm) up to 14 mm, past which conception rates declined. This may suggest increasing rates of endometrial pathology and/or decreased endometrial receptivity beyond a certain threshold, but larger studies would be required to confirm this trend. Our analysis was limited by the relatively smaller amount of cycles in both the lowest (< 7 mm) and highest (≥14 mm), however we believe these to be an accurate representation of the EST distribution within the FSH-IUI population.

The lack of live birth outcome data is also a limitation of this retrospective study. Our institutional research ethics board approved collection of retrospective data from our centre, but live birth rates were not reliably collected for this time period. Further studies with live birth data would augment the findings of our research study.

Although peak EST was associated with cycle outcome using the above described analyses, the lack of significance on ROC curve analysis suggests that EST measurement in isolation is a poor predictor of cycle outcomes. This is similar to the findings of a previous analysis by Kolibianakis et al., that failed to demonstrate the ability of endometrial thickness to predict outcome in CC-stimulated IUI cycles [19].

In conclusion, peak EST has a non-linear relationship with conception outcomes in gonadotropin-stimulated IUI cycles. There is also a trend towards increasing clinical pregnancy rates with increasing peak EST, up to 14 mm, beyond which pregnancy rates diminish. Though our study did not have a high amount of cycles in these groups, there is an indication that both extremes of EST are associated with lower odds of both clinical pregnancy and conception in FSH-IUI cycles.

This non-linear relationship makes it challenging to use peak EST in isolation as a prognostic factor of IUI success. We have demonstrated from univariate and ROC analysis that absolute EST measurement is a poor predictor of outcome. Other than peak EST, clinical pregnancy outcome is associated with other patient and cycle factors including patient age, number of dominant follicles, and total motile sperm count. As an isolated measurement, peak EST has little predictive value, and should therefore not be used as the sole indication for cycle continuation or cancellation. Furthermore, the findings of our study do not support routine cycle cancellation for cycles with a “thin lining” measuring < 7 mm, as clinical pregnancies occur in reasonable frequency under this arbitrary threshold. Similarly, we do not advocate for cancellations for a “thick lining” when EST > 14 mm. However, we recommend that considerations of endometrial pathology be included when EST measurements are in this range.

## Abbreviations

CC: Clomiphene citrate
FSH: Follicle stimulating hormone
GEE: Generalized estimating equation
hCG: Human chorionic gonadotropin
hMG: Human Menopausal Gonadotropin
IUI: Intrauterine insemination EST Endometrial stripe thickness
LH: Luteinizing hormone
ROC: Receiver operating curves
SERM: Selective estrogen receptor modulator
TVUS: Transvaginal ultrasound

## References

[1] Dehbashi S, Parsanezhad ME, Alborzi S, Zarei A (2003). Effect of clomiphene citrate on endometrium thickness and echogenic patterns. Int J Gynecol Obstet.

[2] Birkenfeld A, Beier HM, Schenker JG (1986). The effect of clomiphene citrate on early embryonic development. endometrium and implantation Human Reproduction.

[3] Haritha S, Rajagopalan G (2003). Follicular growth, endometrial thickness, and serum estradiol levels in spontaneous and clomiphene citrate-induced cycles. Int J Gynecol Obstet.

[4] Nakamura Y, Ono M, Yoshida Y, Sugino N, Ueda K, Kato H (1997). Effects of clomiphene citrate on the endometrial thickness and echogenic pattern of the endometrium. Fertil Steril.

[5] Esmailzadeh S, Faramarzi M (2007). Endometrial thickness and pregnancy outcome after intrauterine insemination. Fertil Steril.

[6] Warrington C, Faraj R, Willett M (2008). Endometrial thickness and outcome in sub-fertile women treated with clomiphene citrate. J Obstet Gynaecol.

[7] Gonen Y, Casper RF (1990). Sonographic determination of a possible adverse effect of clomiphene citrate on endometrial growth. Hum Reprod.

[8] Habibzadeh V, Mahani SN, Kamyab H (2011). The correlation of factors affecting the endometrial thickness with pregnancy outcome in the IUI cycles. Iranian journal of reproductive medicine.

[9] Jeon YE, Jung JA, Kim HY, Seo SK, Cho S, Choi YS, Lee BS (2013). Predictive factors for pregnancy during the first four intrauterine insemination cycles using gonadotropin. Gynecol Endocrinol.

[10] Tsai HD, Chang CC, Hsieh YY, Lee CC, Lo HY (2000). Artificial insemination. Role of endometrial thickness and pattern, of vascular impedance of the spiral and uterine arteries, and of the dominant follicle. The Journal of reproductive medicine.

[11] Weiss NS, Van Vliet MN, Limpens J, Hompes PG, Lambalk CB, Mochtar MH, Van Der Veen F, Mol BW, Van Wely M (2017). Endometrial thickness in women undergoing IUI with ovarian stimulation. How thick is too thin? A systematic review and meta-analysis. Hum Reprod.

[12] Yavuz A, Demirci O, Sözen H, Uludoğan M (2013). Predictive factors influencing pregnancy rates after intrauterine insemination. Iranian journal of reproductive medicine..

[13] Revelli A, Poso F, Gennarelli G, Moffa F, Grassi G, Massobrio M (2006). Recombinant versus highly-purified, urinary follicle-stimulating hormone (r-FSH vs. HP-uFSH) in ovulation induction: a prospective, randomized study with cost-minimization analysis. Reprod Biol Endocrinol.

[14] De Geyter C, Schmitter M, De Geyter M, Nieschlag E, Holzgreve W, Schneider HP (2000). Prospective evaluation of the ultrasound appearance of the endometrium in a cohort of 1,186 infertile women. Fertil Steril.

[15] Al-Ghamdi A, Coskun S, Al-Hassan S, Al-Rejjal R, Awartani K (2008). The correlation between endometrial thickness and outcome of in vitro fertilization and embryo transfer (IVF-ET) outcome. Reprod Biol Endocrinol.

[16] Weissman A, Gotlieb L, Casper RF (1999). The detrimental effect of increased endometrial thickness on implantation and pregnancy rates and outcome in an in vitro fertilization program. Fertil Steril.

[17] Israel R, Isaacs JD, Wells CS, Williams DB, Odem RR, Gast MJ, Strickler RC (1996). Endometrial thickness is a valid monitoring parameter in cycles of ovulation induction with menotropins alone. Fertil Steril.

[18] Dinelli L, Courbiere B, Achard V, Jouve E, Deveze C, Gnisci A, Grillo JM, Paulmyer-Lacroix O (2014). Prognosis factors of pregnancy after intrauterine insemination with the husband's sperm: conclusions of an analysis of 2,019 cycles. Fertil Steril.

[19] Kolibianakis EM, Zikopoulos KA, Fatemi HM, Osmanagaoglu K, Evenpoel J, Van Steirteghem A, Devroey P (2004). Endometrial thickness cannot predict ongoing pregnancy achievement in cycles stimulated with clomiphene citrate for intrauterine insemination. Reprod BioMed Online.

